# Kinetic Analysis of Guanidine Hydrochloride Inactivation of ****β****-Galactosidase in the Presence of Galactose

**DOI:** 10.1155/2012/173831

**Published:** 2012-09-13

**Authors:** Charles O. Nwamba, Ferdinand C. Chilaka

**Affiliations:** ^1^Department of Chemistry, University of Idaho, 875 Perimeter Drive, MS 2343, Moscow, ID 83844-2343, USA; ^2^Department of Biochemistry, University of Nigeria, Nsukka, Enugu State 410001, Nigeria

## Abstract

Inactivation of purified **β**-Galactosidase was done with GdnHCl in the absence and presence of varying [galactose] at 50°C and at pH 4.5. Lineweaver-Burk plots of initial velocity data, in the presence and absence of guanidine hydrochloride (GdnHCl) and galactose, were used to determine the relevant *K*_*m*_ and *V*_max_ values, with p-nitrophenyl **β**-D-galactopyranoside (pNPG) as substrate, *S*. Plots of ln([*P*]_*∞*_ − [*P*]_*t*_) against time in the presence of GdnHCl yielded the inactivation rate constant, *A*. Plots of *A* versus [*S*] at different galactose concentrations were straight lines that became increasingly less steep as the [galactose] increased, showing that *A* was dependent on [*S*]. Slopes and intercepts of the 1/[*P*]_*∞*_ versus 1/[*S*] yielded *k*_+0_ and *k'*_+0_, the microscopic rate constants for the free enzyme and the enzyme-substrate complex, respectively. Plots of *k*_+0_ and *k'*_+0_ versus [galactose] showed that galactose protected the free enzyme as well as the enzyme-substrate complex (only at the lowest and highest [galactose]) against GdnHCl inactivation. In the absence of galactose, GdnHCl exhibited some degree of non-competitive inhibition. In the presence of GdnHCl, galactose exhibited competitive inhibition at the lower [galactose] of 5 mM which changed to non-competitive as the [galactose] increased. The implications of our findings are further discussed.

## 1. Introduction 

 A folded protein does not exist in a single conformation, rather as a set of related conformations whose interconversion involves the making and breaking of the weak (noncovalent) interactions such as hydrogen bonds, van der Waals, salt bridges, and hydrophobic interactions that stabilize the folded structure of the protein. The range and speed of interconversions between the conformations will depend on the magnitudes of the relevant energy barriers. In essence therefore, the observed three-dimensional structure of a protein should be viewed as a weighted average of all the conformations accessible on the time scale in question [1] in specific environments. The folding energy landscape theory or the folding funnel concept, used to explain the principle of folding, suggests that the most realistic concept of a protein is a minimally frustrated heteropolymer with a funnel-like rugged energy landscape biased towards the native structure [2–6]. The ruggedness of the energy landscape is biologically essential, controlling the distribution of protein conformations along the biologically relevant landscape, not necessarily around the funnel bottom [7]. The structures of many enzymes are subject to conformational flux (changes/flexibility) during biological function and, thus, conformational fluctuations are coupled to catalysis [8]. The functional properties of enzymes are defined by the same interactions that define stability since they define not only the overall structure of a protein, but also the presence and location of regions with different propensities to undergo conformational rearrangements [9]. Thus, the stability of a protein depends on protein structure and function. The protein folding problem (Levinthal's paradox) deals with understanding how a protein searches its conformational space so quickly, attaining its native conformation within a very short period of time (microseconds or less) amidst the array of vast alternative conformers within its search frame [10] and, by implication, can be connected to protein stability [11] since the attained state is marginally stable compared to all other feasible conformations. Protein denaturation remains the primary source of information on the structural energetics of globular proteins and provides test data from which the contributions of the various interactions that stabilize the protein structure/function can be determined [12–14]. 

Unfolded peptides, polypeptides, and chemically unfolded proteins are flexible [15–17]. Flexible molecules exhibit conformational diversity. The more flexible the protein, the larger the ensemble of conformers. Such proteins can bind to a range of potential ligands and can be pictured as having a very rugged funnel bottom with rather low (induced fit) or high kinetic barriers (conformational selection) separating the multiple minima valleys [18, 19]. The conformer that binds a ligand is the one that is complementary to it, with the conformational equilibrium adjusting in favour of this conformer. While one conformation fits one ligand, an alternate conformer may be more favourable for binding a ligand with a different structure [20]. All these are mediated by changes in the environment of the molecule [19]. Thus, molecular flexibility enables the protein to bind to a range of potential ligands [18]. 

Ligand binding not only increases the rate at which denatured enzymes regain their activity during renaturation in their presence, but also maintain the native conformation of proteins during denaturation in their presence. This suggests that ligands act as a folding nucleus about which the remaining constructed regions are easily induced to assume a more biologically active conformation [21]. Recently, it has been suggested that ligands especially inhibitors can function as molecular chaperones [22]. Furthermore, there is also no doubt that a two-state binding process, in which binding and folding take place simultaneously, also displays a funnel-like shape [18]. The funnel arises because the drive towards a hydrophobic collapse (as in protein folding) is also a drive toward a reduced ensemble of conformations (as in both folding and binding where one conformation generally predominates) [5]. The binding of a ligand to a denatured protein could thus lead to the refolding of the protein with accompanying enzymatic activities [23]. In both folding and binding, the processes initiate from a higher energy and terminate in lower energy states, regardless of the pathways that are followed [18]. Folding and binding are connected by a common parameter: the energy landscapes. 

We have recently shown that the conformational isomer taken up by a ligand-induced folding of a protein during denaturation is a function of the ligand type (product inhibitor) and concentration [23, 24]. We also showed that the galactose-induced refolding of *β*-galactosidase in the presence of urea was effected via different inhibition patterns. While folding is modulated by the solvent environment [25, 26], the peculiar binding energetics of an amino acid sequence in an unfolded polypeptide could enable the polypeptide attain well-defined structures [17]. We employed the analysis of Tian and Tsou [27], who suggested that, from the effect of [*S*] on *A*, noncompetitive inhibition is involved when *A* is independent of [*S*], while a straight line will be obtained either in the plot of 1/*A *against [*S*] for competitive inhibition, or 1/*A *against 1/[*S*] for uncompetitive inhibition. Alternatively, from the effect of [*S*] on [*P*]_∞_, a competitive inhibition is predicted when a plot of [*P*]_*∞*_ against [*S*] gives a straight line passing through the origin. For noncompetitive inhibition, the plot of 1/[*P*]_*∞*_ against 1/[*S*] will be a straight line whereas for uncompetitive inhibition [*P*]_*∞*_ will be independent of [*S*]. In this work, we study the binding of galactose to *β*-galactosidase in the presence of the denaturant, GdnHCl, and end with a summary of the import of our findings to the current knowledge of protein folding. Besides, we also contrast the relevance of our present findings from our previous work [23].

**Theory**

**Scheme 1:**
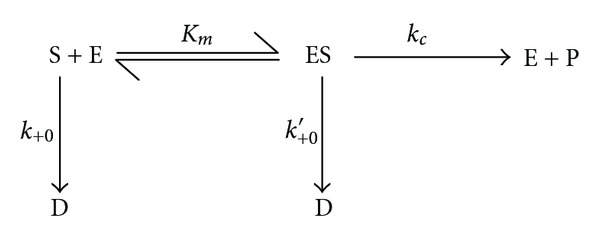
*E*, *D*, *K*_*m*_, *k*_*c*_, *k*_+0_, and *k*_+0_′ represent the native enzyme, denatured enzyme, the Michaelis constant, the turnover number of enzyme catalysed reaction in the presence of denaturant, the first order microscopic rate constant for the free enzyme, and the first order microscopic rate constant for the enzyme-substrate complex, respectively. All the kinetic constants are functions of the denaturant concentrations and thus functions of the apparent inactivation rate constant, *A*.

The kinetic analysis of the effects of substrate concentration on GdnHCl inactivation of *Kestingiella geocarpa β*-galactosidase in the presence and absence of galactose was a combination of the procedures of Xiao et al. [28] and Wang et al. [29]. The scheme of enzyme inactivation by denaturants in the presence of the substrate is as shown in Scheme 1 while the subsequent derivation of parameters and calculations relating enzyme inactivation by denaturants to product (P) formation at given time intervals *t* is as shown by Chilaka and Nwamba [23].

## 2. Materials and Methods

### 2.1. Materials

Fresh, dry, unwrinkled, and mature (*Kestingiella geocarpa*) seeds were bought from the Nsukka (Nigeria) main market. p-Nitrophenyl *β*-D-galactopyranoside (pNPG) and guanidine hydrochloride (GdnHCl) used were purchased from Sigma Chemical company (St. Louis, MO, USA) and BDH (England), respectively. All other reagents used were of Analar grade.

### 2.2. Germination of Seeds

The seeds of *Kestingiella geocarpa *were germinated as already described in Chilaka and Nwamba [23].

### 2.3. Enzyme Extraction and Purification

The enzyme was extracted and purified according to the method of Chilaka et al. [24].

### 2.4. Protein Estimation

Protein concentration was determined by the method of Lowry et al. [30].

### 2.5. Enzyme Assay

Assay for enzyme activity after purification was carried out as described by Chilaka et al. [24].

### 2.6. Effect of Substrate (pNPG) Concentration on GdnHCl Inactivation of *β*-Galactosidase in the Presence and Absence of Galactose

The method of assay for the enzyme activity in the presence and absence of galactose is as already described by Chilaka et al. [24]. However, in this instance, the substrate concentration ranged from 0.10 mM to 0.60 mM. The [galactose] employed in the study was from 5–20 mM. Briefly, the substrate, pNPG, and/or denaturant or substrate, denaturant and varying [galactose] were incubated at 50°C for 10 minutes in 0.10 M sodium acetate buffer, pH 4.5, while the enzyme was also incubated in a separate test tube, in the same buffer and at the same temperature. Substrate, denaturant, and galactose concentrations were calculated based on the total volume of the reaction vessels on introduction of the enzyme. Prior to the start of the experiment, aliquots were pooled off from the setup not containing the enzyme. This served as the blank. The reaction was started by introducing the enzyme into the test tube containing the substrate and/or denaturant or substrate, denaturant and galactose (all in the sodium acetate buffer) and one mL aliquots pooled off at varying time intervals and introduced into 4 mL NaOH (0.10 M) to stop the reaction and develop colour. The absorbance of the solution was measured at 400 nm and the concentration of p-nitrophenol released read off a p-nitrophenol standard curve. One unit of activity is the amount of enzyme liberating 1 mmol of p-nitrophenol per minute.

## 3. Results 

 Lineweaver-Burk plot of initial velocity data of the native enzyme in the absence of Guanidine hydrochloride (GdnHCl) gave a *K*_*m*_ of 0.25 mM and a *V*_max⁡_ of 15.48 *μ*mole/minute; while galactose was a competitive inhibitor with a *K*_*i*_ of 26.0 mM [24].

**Figure 1:**
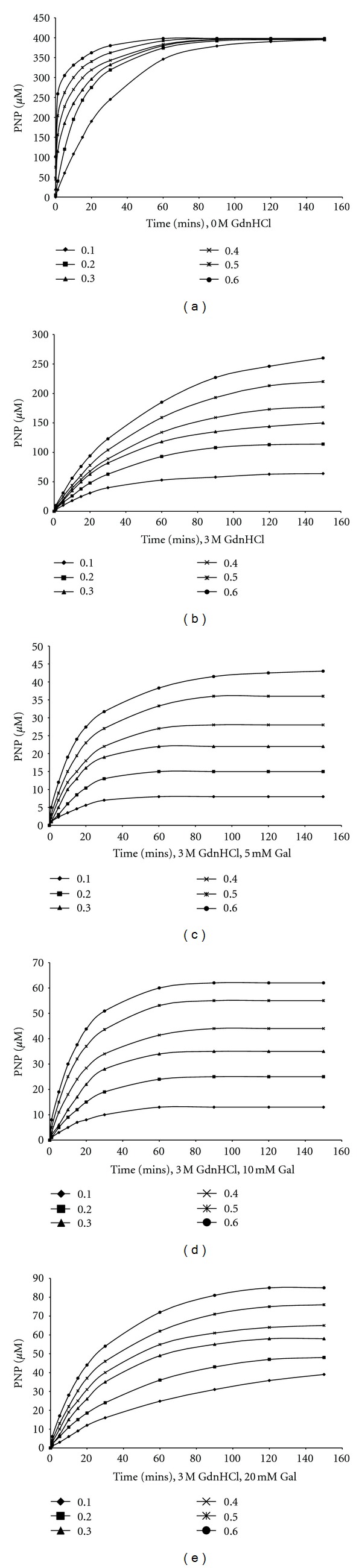
Kinetics of the inactivation of *β*-galactosidase in the absence and presence of 3 M GdnHCl at 50°C, pH 4.5, and at different concentrations of substrate, PNPG, [0.10–0.60]. (a) In the absence of GdnHCl (no GdnHCl); (b) in the presence of 3 M GdnHCl; (c) in the presence of 3 M GdnHCl and 5 mM galactose; (d) in the presence of 3 M GdnHCl and 10 mM galactose; (e) in the presence of 3 M GdnHCl and 20 mM galactose.

**Figure 2:**
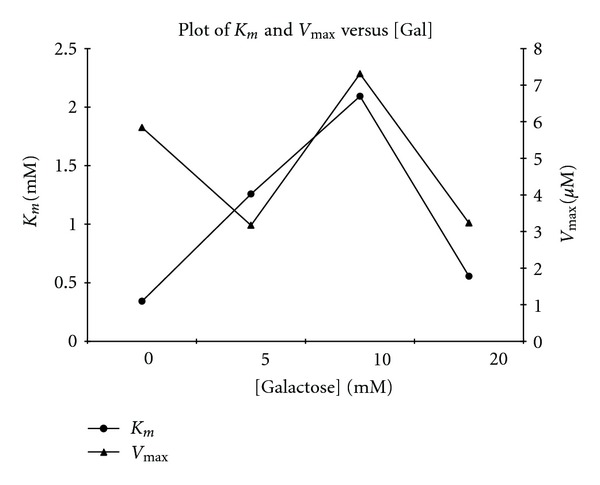
Effect of GdnHCl and galactose on the *K*_*m*_ and *V*_max⁡_ of *β*-galactosidase using pNPG as substrate, both in the presence and absence of galactose. *K*_*m*_ and *V*_max⁡_ values were calculated from Lineweaver-Burk plots of initial velocity data at the concentrations of GdnHCl and galactose indicated.

 Time curves (plots of [*P*]_*t*_ (p-nitrophenyl released versus the time, *t*) were plotted for 0, 3 M-GdnHCl (Figures 1(a) and 1(b)) in the absence of galactose, and for 3 M GdnHCl in the presence of 5 mM, 10 mM and 20 mM galactose, respectively, (Figures 1(c)–1(e)). The results showed that the concentration of product, [*P*] ([pNP]) formed at any time interval *t*, was directly related to the substrate concentration, [*S*] ([pNPG]). With increase in reaction time *t*, [*P*]_*t*_ approached a constant value [*P*]_∞_, at each [pNPG]. However, in the presence of the 3 M GdnHCl, the [*P*]_∞_ for each [*S*]_*o*_ decreased with respect to that in the absence of the [GdnHCl] (Figures 1(a) and 1(b)). On introduction of the 5 mM galactose, [*P*]_∞_ decreased drastically (Figure 1(c)), but the [*P*]_∞_ dramatically increased through 10 mM to 20 mM galactose (Figures 1(d) and 1(e)). However, at 20 mM galactose, the [*P*]_∞_ formed for the highest [*S*]_*o*_ was still much lower to that formed in the presence of the GdnHCl alone. Actually, the [*P*]_∞_ for 3 M GdnHCl alone at [*S*]_*o*_ of 0.60 mM was over three times more than that formed when 20 mM galactose was introduced. 3 M GdnHCl alone caused the *K*_*m*_ to increase and *V*_max⁡_ to decrease with respect to its absence. When the various [galactose] were introduced in the presence of the GdnHCl, the *K*_*m*_ increased for 5 mM–10 mM and decreased for 20 mM galactose. The *V*_max⁡_ from 5–20 mM galactose also followed this trend except the 5 mM that had the lowest value compared to GdnHCl alone (Figure 2). Interestingly, the various *K*_*m*_ in the presence of the [galactose] were all higher to that of the GdnHCl alone; while with the exception of the 10 mM galactose, the *V*_max⁡_ for the 3 M GdnHCl was higher than those in the presence of galactose. 

**Figure 3:**
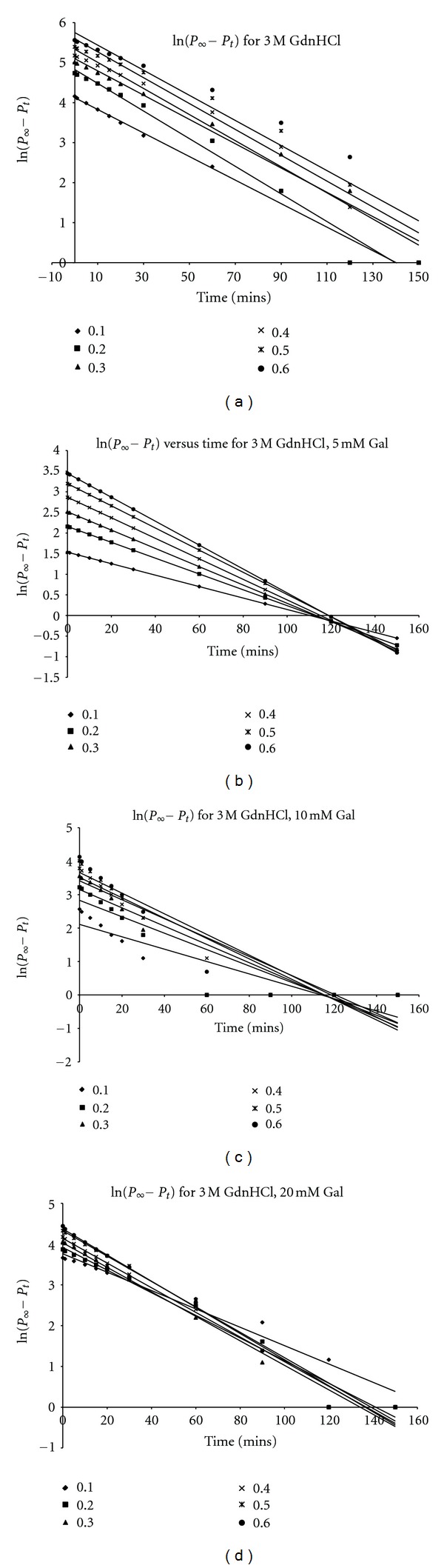
Semilogarithmic plot of P(*μ*M) versus time (*t*) of data: (a) ln([*P*]_∞_ − [*P*]_*t*_) versus *t* for 3 M GdnHCl, 0 mM galactose; (b) 3 M GdnHCl, 5 mM galactose; (c) 3 M GdnHCl, 10 mM galactose; (d) 3 M GdnHCl, 20 mM galactose. N.B: [*P*]_*∞*_ = [pNP]; [*P*]_*t*_ = [pNP]*t*.

**Figure 4:**
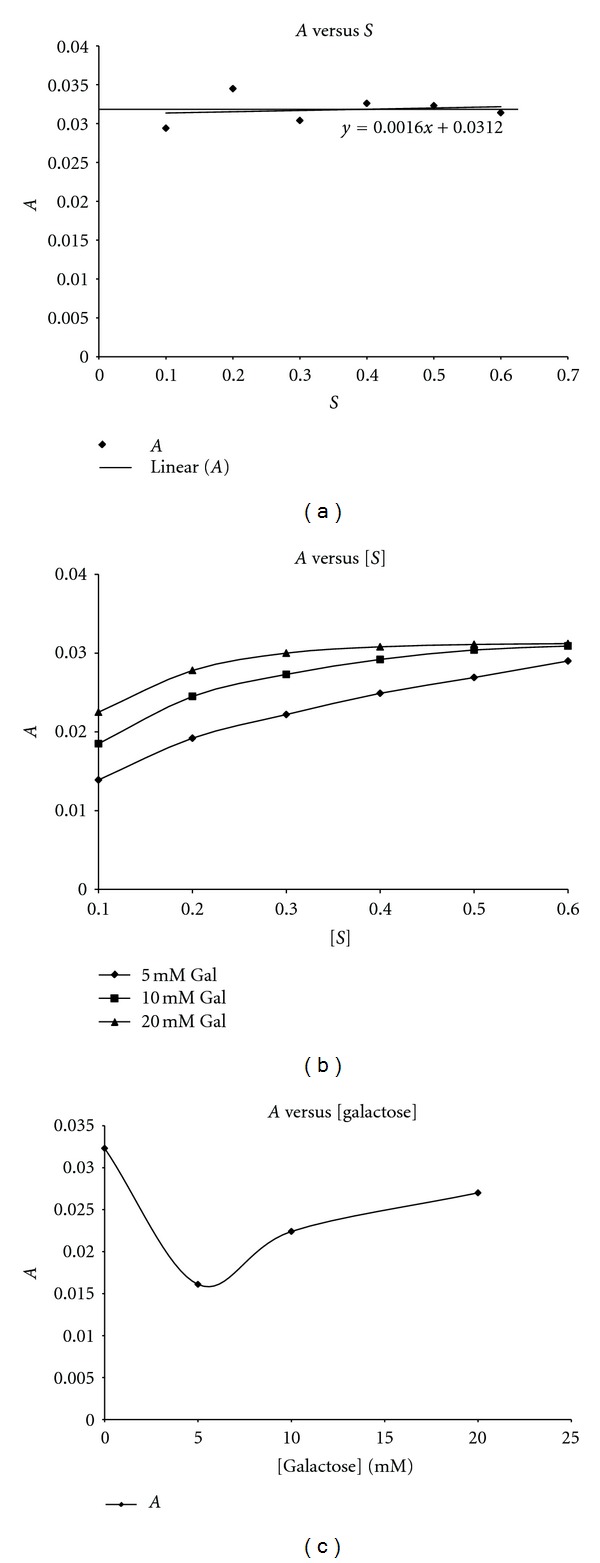
Plot of apparent inactivation rate constant *A* against substrate (pNPG) concentration. (a) In the presence of 3 M GdnHCl only; (b) in the presence of 3 M GdnHCl and all the [galactose]; (c) *A* versus [galactose].

**Figure 5:**
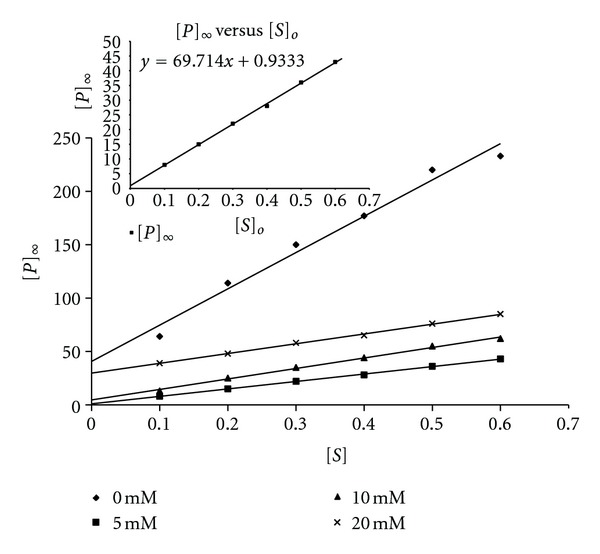
Plots of [*P*]_*∞*_ against [*S*] for *β*-galactosidase in the presence of 3 M GdnHCl—5 mM galactose, 3 M GdnHCl—10 mM galactose, and 3 M GdnHCl—20 mM galactose. Inset is the magnification of the graph of [*P*]_*∞*_ versus [*S*] for 3 M GdnHCl—5 mM galactose showing that the intercept on the *y*-axis is slightly above the zero point.

Plots of ln([*P*]_*∞*_ − [*P*]_*t*_) versus time *t* (Figure 3) gave straight lines (first order kinetics) with slopes corresponding to *A*, the apparent inactivation rate constant. For ease of calculation and plotting, regression analysis was employed to calculate the slopes of the ln([*P*]_*∞*_ − [*P*]_*t*_) versus time plots. Plots of *A* versus [*S*]_*o*_ in the absence of galactose was zero order (Figure 4(a)), showing that the substrate had no protective effect on the enzyme inactivation. The value of *A* for the 3 M GdnHCl was 0.0323 s^−1^. In the presence of galactose, plots of *A* versus [*S*]_*o*_ showed that *A* was dependent on [*S*]_*o*_, with a positive slope, which became increasingly less steep from 5 mM to 20 mM (Figure 4(b)). Experimentally, the type of inhibition can be ascertained by studying the effect of [*S*] either on the apparent rate constant, *A* or on [*P*]_*∞*_ [27]. The plot of *A* against [galactose] shows that 3 M GdnHCl alone (i.e., 0 mM galactose) had the highest *A* value when compared to those for the [galactose]. However, with respect to the [galactose], the value of *A* rose slightly from 5 mM through 10 mM to 20 mM (Figure 4(c)). Plots of [*P*]_∞_ versus [*S*] (Figure 5) gave straight lines passing near to the origin for the 5 mM galactose (see insert in Figure 5). This indicated that the [galactose] was exhibiting a near competitive inhibition at the lower [galactose] of 5 mM which changed to non-competitive as the [galactose] increased. The high intercept on the *y*-axis of the plot of [*P*]_∞_ versus [*S*] for the 3 M GdnHCl alone indicates that the GdnHCl exhibited some degree of non-competitive inhibition on the enzyme.

**Figure 6:**
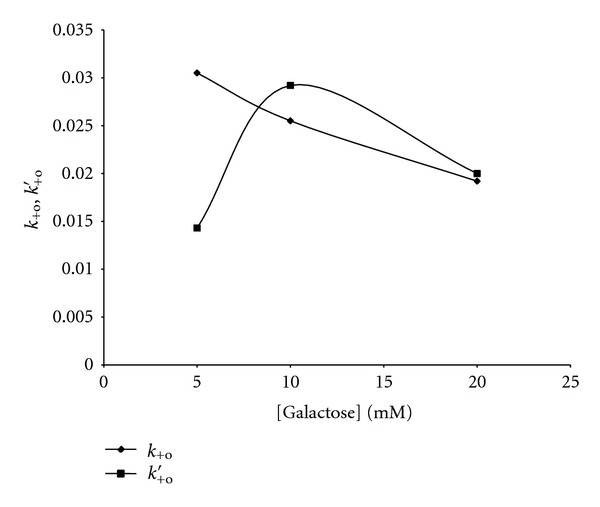
Plot of *k*_+o_, *k*_+o_′ versus [galactose]. *k*_+o_ and *k*_+o_′ are the microscopic inactivation rate constants for the free enzyme and enzyme-substrate complex, respectively.

 Plots of 1/[*P*]_∞_ versus 1/[*S*]_*o*_ at different [galactose] yielded *k*_+o_ and *k*_+o_′. Plots of *k*_+o_ and *k*_+o_′ versus [galactose] (Figure 6) showed that *k*_+o_ versus [galactose] was a hyperbolic curve decreasing from 0.0305 s^−1^ to 0.0192 s^−1^, while *k*_+o_′ versus [galactose] gave a hyperbolic curve with *k*_+o_′ increasing from 0.0143 s^−1^ to 0.0292 s^−1^ and then decreasing to 0.0200 s^−1^ at [galactose] of 20 mM. This demonstrated a protection of the free enzyme as the [galactose] increased while the enzyme-substrate complex was protected, most especially, at the lowest and highest [galactose], respectively. 

## 4. Discussion

During chemical or physical denaturation of many enzymes, inactivation may or may not parallel overall conformational changes [28]. With urea as the denaturant of *β*-galactosidase from *K. geocarpa *[23], it was suggested that inactivation occurred before measurable conformational changes, with the dominant inactivation/denaturation pathway involving changes in the enzyme active site. By employing a similar analysis, we have investigated the effect of GdnHCl on the kinetics of unfolding/refolding of *β*-galactosidase in the presence and absence of galactose. 

In the presence of GdnHCl, the [*P*]_∞_ at all [*S*]_*o*_ decreased in comparison with the absence of the denaturant. This demonstrates the denaturing effect of the GdnHCl on the enzyme activity. The 5 mM galactose drastically lowered the [*P*]_∞_ of the enzyme with respect to that in the presence of the GdnHCl and absence of galactose (0 mM galactose). At this concentration of galactose as determined from the plot of *k*_+o_, *k*_+o_′ versus [galactose], the dominant inhibition mechanism is the binding of the galactose to the enzyme via a competitive mode of inhibition. Characteristic of competitive inhibition was an increase in *K*_*m*_, while the decrease in *V*_max⁡_ shows that the inhibition mechanism was not wholly competitive. A reviewer of this paper brought to our notice a possibility that our results could also be due to nonproductive binding variant of competitive inhibition.

Compared to the 5 mM galactose, the 10 mM galactose had a higher partition ratio, *r* ([*P*]_∞_/[*E*]_*o*_) and the highest *K*_*m*_ and *V*_max⁡_ values. Also at 10 mM galactose, there was an enhanced non-competitive inhibition mechanism when compared to the 5 mM galactose as seen from the intercepts on the *y*-axis of the plots of [*P*]_∞_ versus [*S*]_*o*_. One consequence of a high *K*_*m*_ is decrease in substrate specificity and decreased binding affinity, as a result of unstructuredness and increased flexibility of the enzyme active site induced by the GdnHCl. When *K*_*m*_ > [*S*]_*o*_, the enzyme binds the substrate weakly and, therefore, the [ES] is present only at low concentrations. It has been suggested that, for the low *K*_*m*_ binding processes with the tightly bound substrate, the ground state of the reaction is the ES complex, and the activation energy of the reaction is higher than for the high *K*_*m*_ binding processes involving a weakly bound substrate in which the ground state of the reaction is the free reactants [31]. Thus, a low-energy enzyme-substrate complex is a “thermodynamic pit,” from which the reaction has to climb out [31]. As high *K*_*m*_ binding processes are incompatible with accumulation of intermediates but have the goal of maximizing the reaction rate, the predominant competitive inhibition mechanism at 5 mM galactose where *K*_*m*_ > [*S*]_*o*_ would prevail over the rate of product formation when compared with 10 mM galactose which was less competitive and more non-competitive. The 20 mM galactose exhibited, essentially, a non-competitive inhibition mechanism. As could be deduced from the plot of *K*_*m*_ or *V*_max⁡_ versus [galactose], the 10 mM galactose signified the transition between a predominantly competitive (below 10 mM) and a predominantly non-competitive (above 10 mM) inhibition pattern. Although the 10 mM galactose had a higher *V*_max⁡_ compared to the 20 mM galactose, the 20 mM still had a higher partition ratio, *r*, compared to the 10 mM galactose. This is not surprising since the galactose appears to enhance the effect of the GdnHCl on the free enzyme, although this effect appears inversely proportional to the [galactose] (see *k*_+o_ column in Table 1). While the *A* values from 5–20 mM rose steadily, the *k*_+o_ and *k*_+o_′ values decreased correspondingly from 5–20 mM galactose except for the rise in the *k*_+o_′ value for 10 mM galactose (Table 1). In fact at 20 mM, the galactose not only protected the free enzyme, but also conferred some protection on the [ES] with respect to that of the 10 mM galactose. Probably, by binding and accumulation within the region of the active site (but not catalytic active site), the galactose was locally protecting the free enzyme and, partly, the enzyme-substrate complex, via a predominantly non-competitive inhibition mechanism, while the whole enzyme molecule was still being globally destroyed by the denaturant. In other words, as the galactose concentration decreased from the region of the enzyme active site, most probably to the interior of the molecule, the level of unstructuredness induced by the denaturant on the molecule increased. The 3 M GdnHCl alone (absence of galactose) had the least *k*_+o_′ value, as well as having a *k*_+o_ value higher than that of the 20 mM galactose, yet it had the highest *A* value when compared to those of the 5–20 mM galactose. This would indicate that, even though the substrate, pNPG, might confer about two times greater protection to the [ES] complex compared to the free enzyme ([*E*]_*o*_) in the absence of galactose, however, it could not induce native structural reorganization that favored the native state formation. Furthermore, the plot of *A* versus [*S*] for the GdnHCl was a zero order plot showing that on a global scale the substrate could not protect the enzyme; rather, it did so only at a local level (within the active site region).

 In relation to the energy landscape, binding of the [*S*] leads to catalysis via the modulation of the binding funnel towards reduced energy states with lower entropy, but with no apparent effect on the folding funnel. In other words, within the conformational equilibrium induced by the 3 M GdnHCl, the substrate binds and displaces the equilibrium towards a conformer or conformers with appropriate catalytic activity without the need to initiate global refolding. In the case of galactose, in an apparent paradox, even though the galactose (up to 10 mM) potentiated the inhibitory/inactivating effect of the GdnHCl on catalysis probably by synergism, it simultaneously modulated folding to a reduced ensemble of states as was seen by the decreased *A* with respect to the absence of galactose and 3 M GdnHCl. Above 10 mM, the full folding potential of galactose was realized, despite its inhibitory ability. Thus, a bit of catalytic proficiency was sacrificed for folding.

 GdnHCl and urea perturb proteins by disintegrating the bonds needed to maintain the 3-dimensional (native) structure of the molecule. This leads to an uphill rise in the energy level of the molecule and an increase in its conformational entropy. Even though the number of protein conformations and potential binding sites grow dramatically with increasing steps up the energy ladder, Boltzmann's law dictates that the ligand prefers to choose from the relatively few ligation states low on the energy ladder [32]. Thus, even though high conformational entropy dictates a high number of energetically accessible states within a topology space, only a limited number of these states are energetically preferred [33]. As the bottom of the energy ladder narrows (the funnel concept), only molecules of low dimensionality and size would most probably bind to the few binding sites. Galactose, being able to fit more into the enzyme active site during catalysis of either the synthetic substrate, pNPG, or the natural substrate, lactose, would easily bind to different conformers of the enzyme when compared to the whole substrate molecule (pNPG) (or even lactose). Both pNPG and/or even lactose, due to greater bulk and thus, steric hindrance, would be limited in fitting into *available* enzyme conformers to modulate both binding and folding via shifts in the dynamic energy landscape which is a common denominator to both binding and folding. Thus, pNPG would not easily couple binding and folding as would galactose. In energy terms, galactose would readily bind to the low energy binding site(s) (as a function of concentration) to drive catalysis (via binding) and folding (since both binding and folding are geared towards a reduced ensemble of states), while pNPG would bind somewhere up the energy ladder to bring about a kinetic shift [19] to a local minimum that can only favour catalysis without been coupled to folding. The similarity of binding and folding is clear at the thermodynamic level, where both processes involve accurately locating molecular fragments with respect to each other, reducing the configurational entropy, and simultaneously lowering the free energy by the exclusion of solvent and formation of hydrogen bonds and salt bridges [34–36]. Thus, it could be understood why increasing [galactose] would have to wade through the sea of atoms of the enzyme molecule to the interior of the protein. 

 The action of the GdnHCl and urea creates some ruggedness around the bottom of the funnel (both binding and folding) [19, 32] that depicts various conformers. Ligand-induced isomerization [37] of the various enzyme conformers could be induced by galactose around the funnel bottom via different binding modes of inhibition modulated by the environment of the enzyme. This conformational reorganization around the funnel bottom is likely to be largely enthalpic rather than entropic [38], involving mainly residues backbone reorganization [39]. The ligand by binding to these conformers modulates the population shifts to redistribute around the predominating conformer (reviewed in [19]). The energy landscape which is dynamic could be shifted to favour a minimum with the population reequilibrating to that minimum, via an intermediary step populated mostly by secondary structural interactions and few tertiary interactions—the molten globule [23, 40–44]. Additionally, since the denaturants create the ruggedness in the funnel, then energetic frustration becomes a key factor in the coupled binding and folding mechanism. However, the ligand by selecting a conformation as modulated by its concentration eases off the ruggedness of the funnel so that minimal frustration accompanies the subsequent folding. From the plot of *K*_*m*_ versus [galactose], it is clear that the various conformers have a high kinetic barrier between them since their *K*_*m*_ *S* vary considerably. The ligand as a function of its concentrations binds to conformers most precise to a given concentration (conformational selection) and “pulls” the equilibrium via the dynamism of the energy landscape to favor the given conformer. We also suggest that since the *k*_+o_ and the *k*_+o_′ decreased as the mode of inhibition became increasingly non-competitive (i.e., as the location of binding tilted more towards the hydrophobic core) while the *A* increased, then there must be some sort of induced fit mechanism which propagates from the site of binding [9, 45] of the galactose to the exterior of the molecule so that as the galactose moves progressively inwards, the propagation of induced fit to the exterior diminishes and thus *A* increases irrespective of the decreasing *k*_+o_ and *k*_+o_′. Thus, galactose (unlike pNPG) effectively coupled binding to folding (so that folding occurred during binding), a trait characteristic of intrinsically unfolded or disordered proteins [46–48]. One selective advantage of folding only at the time of binding is the possibility to achieve high specificity with low affinity [35, 49]. The [galactose] modulates specificity in the enzyme conformer that is selected, while the high *K*_*m*_, as already discussed, tries to modulate catalysis so as to maximize rate by discouraging the accumulation of intermediates. 

Urea, H_2_N–CO–NH_2_ and GdnHCl, H_2_N–CNH–NH·HCl, are known protein denaturants, but each induces different binding modes of inhibition. A comparison of the effect of urea [23] and GdnHCl (present communication) shows that the binding modes of inhibition are very opposite at the same concentrations of galactose. From the results of Scholtz et al. [50] on some model peptides, the interaction between urea and peptide groups account for a major part of the denaturing action of urea on proteins, and not by the interaction between urea and hydrophobic groups as earlier suggested [34]. Little wonder the dominant inactivation/denaturation pathway using urea on *β*-galactosidase from *K. geocarpa* involved changes in the enzyme active site, which of course is surface located. Thus, for a full protection/reactivation of the enzyme at high [galactose], the dominant binding mode of inhibition would have to involve competitive inhibition. In the case of GdnHCl, in addition to possessing amino (as does urea) and imido groups, the denaturant possesses HCl which is strongly electrostatic/polar (however, since GdnHCl is a salt, it ionizes in solution as the GuH^+^ and Cl^−^ with the GuH^+^ are the more potent charged group). Tsai and Nussinov [51] analyzed 294 salt bridges from a nonredundant data set of 38 high resolution (≤1.6 Å) crystal structures of dissimilar monomeric proteins. They found out that the majority (greater than three-quarters) of the salt bridges are formed within the hydrophobic folding units (domains). Thus, GuH^+^ being strongly electrostatic would disorganize salt bridges in the core of the protein so that the dominant inactivation/denaturation pathway using GdnHCl would involve the hydrophobic core of the protein. Moreover, some thirty-eight years back, Greene and Pace [52] reasoned that since GdnHCl was 2.8 times more effective than urea (which is uncharged though polar) in unfolding ribonuclease but only 1.7 times more effective for lysozyme, then the more polar but buried polypeptide chain of the ribonuclease would have accounted for the greater denaturing capability of the GdnHCl on the ribonuclease to the lysozyme. However, since the dependence of conformational stability (Δ*G*_*D*_) on GdnHCl concentration, *δ*(Δ*G*_*D*_)/*δ*(GdnHCl), increases markedly as the denaturant concentration increases, then this indicates that an increase in the number of GdnHCl binding sites on unfolding is the major driving force for denaturation by GdnHCl [53]. Thus, it could be deduced as earlier proposed by Robinson and Jencks [54] and supported by further experimental works [55] that the strongest binding sites for GdnHCl or its ions on a protein molecule are the aromatic side chains and pairs of adjacent peptide groups by hydrogen bonding with the carbonyl groups. Thus, a proposed model for the denaturation of guanidinium-like species can be said to involve two processes: one, the disruption of water structure and the loosening of hydrophobic interactions and, the other, the solubilization of the interior of the protein due to specific interactions with the peptide bonds and solubilization of the hydrophobic regions [56]. Some years latter, Monera et al. [57] would suggest that since the masking effect of GdnHCl (at low concentrations) on electrostatic interactions (repulsions/attractions) that might be present in the protein would serve to underestimate the electrostatic contribution to stability, then the estimates of protein stability from GdnHCl denaturation studies would likely be a relative measure of the contributions of hydrophobic interactions. Consequently, measurable conformational changes would occur before enzyme inactivation during the enzyme denaturation. This might explain why the 3 M GdnHCl had the highest [*P*]_∞_/[*S*]_*o*_ ratio any given time interval, *t*, in comparison to the presence of the galactose, while still possessing the highest *A*. Even though our *A* values of 0.0161 s^−1^–0.0323 s^−1^ compare favourably well with a value of 0.016 s^−1^ for papain, unlike in the work of Tian and Tsou [27] we cannot rule out a possible inactivation of the enzyme by inhibition from GdnHCl. The plot of [*P*]_∞_ versus [*S*]_*o*_ for the 3 M GdnHCl shows that the GdnHCl was interacting via a non-competitive binding mode with the enzyme that is away from the enzyme active site. However, from the nature of the slope of the [*P*]_∞_ versus [*S*]_*o*_ plots for the GdnHCl alone, in comparison, to those in the presence of galactose, it becomes clear that site specific binding might not have been the only possible mode of binding for the GdnHCl. It is suggested that the solvent-exchange mode of interaction, in which the interactions of both the solvent (buffer) and the cosolvent (GdnHCl) with the protein involve the interchange between the components at a particular interaction “site” on the protein [50, 58] could be operational. As the main site of interaction of the GdnHCl with the protein is the hydrophobic core of the protein, the solvent-exchange system is inevitable. 

We earlier noted the moonlighting properties of denatured *β*-galactosidase to model an intrinsically unstructured protein (IUP) via the modes of inhibition leading to reactivation using galactose [23]. Unlike conventional IUPs which use different sites for binding of the restructuring ligand and catalysis of its substrates to products, respectively [59], this protein could use either the same or different sites for both binding (and subsequently folding) and catalysis. While IUPs are involved in metabolic regulation [46, 47, 59], *β*-galactosidase is also involved in metabolic regulation during seed germination and fruit ripening [60–62]. 

**Scheme 2:**
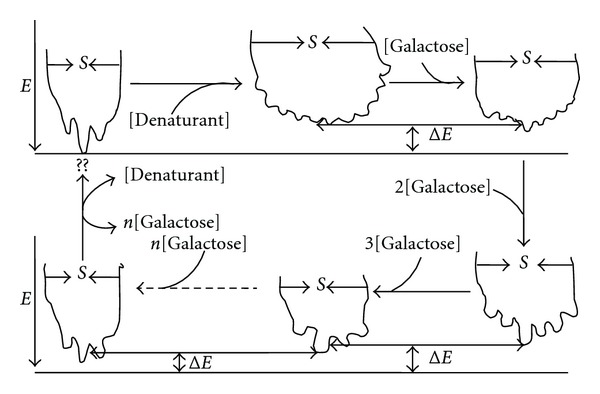
The modulation of conformational substates by the galactose concentrations. *E* is energy, *S* is entropy, and Δ*E* is energy difference between two predominant conformational substates.

It is seen from Scheme 2 above that the denaturant creates some ruggedness around the funnel bottom, corresponding to different substates some of which would lead to misfolding of the protein where it (protein) is to be trapped in those substates. As the galactose is introduced, there is a decrease in the ruggedness on the folding funnel via a decrease in energy, entropy, and the number of the conformers (corresponding to different substates) with one conformer being predominant over the others. As [galactose] increases, the substates become fewer in number and even more distinct with the dominant conformation being all of the time more pronounced and with a lower energy to others around it. On removal of the [galactose] and [denaturant], the protein returns to the native state although in most cases the reformation of a native conformation is extremely slow or even impossible especially when the conformational changes are coupled to ionization reactions. It is seen that the [galactose] concentration modulates the enzyme forms present. The different enzyme forms bind to the substrate. The enzyme forms that bind *S* apparently have the least Δ*G* at a given [galactose] in a local minimum which topologically favours the formation of a native state as a result of coupled binding and catalysis, as mediated by the ligand nature and concentration.

Below is a scheme suggesting the interconvertibility of the binding mode of *β*-galactosidase via the ligand concentration when unstructuredness is induced by a denaturant such as urea or GdnHCl. 

**Scheme 3:**
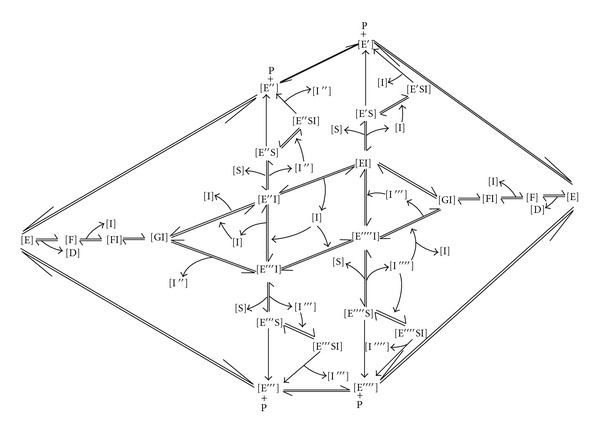
Whereby E is the free/active enzyme form, ES, the enzyme-substrate complex, D is denaturant such as urea or GdnHCl; F, an inactive urea denatured enzyme form; G, an intermediate enzyme form; S, substrate. I′, I′′, I′′′, and I′′′′ are increasing concentrations of ligand (e.g., galactose), **E** ^*n*^ **S** is enzyme-substrate complex whereby *n* represents various conformational states of enzyme bound to substrate, **E** ^*n*^ **I** is enzyme-inhibitor complex whereby *n* represents various conformational states of enzyme bound to inhibitor and P, product, **E** ^*n*^ **S** **I** is enzyme-substrate-inhibitor complex whereby *n* represents various conformational states of enzyme bound to substrate and inhibitor complex.

From Scheme 3, it is seen that the [galactose] (as well as the nature of the ligand [24]) modulates the enzyme forms present. Thus, different enzyme forms bind to the substrate at different [galactose]. The enzyme forms that bind *S* are the enzyme forms that apparently have the least Δ*G* at a given [galactose] in a local minimum which topologically favours the formation of a native state as a result of coupled binding and catalysis, as mediated by the ligand nature and concentration.

 We end our write-up with a summary of the relevance of our findings to the current knowledge of protein folding just as we contrast the relevance of our present findings from our previous work [23].

- Even though urea and GdnHCl are denaturants with some similarities in constituents, both refereed *opposite *binding and inhibition modes at same [galactose]. They do this basically by their interactions with the protein and varying [galactose], which results in different *forces*. It is these forces that determine the outcome of the interactions reflected as different inhibition mechanisms (see [10]). The contrasts between urea and GdnHCl actions highlight the importance of environment in determining the fate of a protein—from folding through functions, kinetics, and thermodynamics to denaturation and breakdown. This work illustrates the subtleties played out by various metabolites in the body and how these players might be the ultimate regulators between health and disease especially in the protein mis-folding diseases such as sickle-cell disease where the conundrum that triggers polymerization and crisis is not immediately known [63, 64]. It is possible that a subtle flux in environmental conditions could be the fine control (besides other fine controls such as epigenetics) and conformational gate keeper to polymerization.
- From our previous [23, 24] and present results, we deduce that both the natures of the denaturants and ligands, as well as the ligand concentrations at any given instance, were responsible for the mode of inhibition at given denaturant and ligand concentrations.
- Our present work suggests that hydrophilic interactions, even within hydrophobic folding cores, contribute substantially to the folding and stability of proteins. This is because the action of GdnHCl differed from urea by perturbing hydrophilic interactions within the protein cores. If these interactions within the folded core were of no importance to folding, stability, and function of the protein, then the inhibition mechanism and kinetics induced by urea and GdnHCl interactions with varying [galactose] would have been same or very similar to each other (see [10, 51] and some other sited references).
- Our results suggests that a so-called *global* minimum is not required for protein function. Once a protein has attained a minimum (local or global) and can function in its current state, then its attained Gibb's free energy for that state becomes its minimum even if it is in a metastable state. Thus, in vivo, many conformers of an enzyme may exist within a conformational/metabolic enclave, so that subtle environmental factors become the important parameters in a selection process making “the native state” a relative term and idiosyncratic.
- Jaffe's group [65] suggested that from our previous work [23], *β*-galactosidase probably exhibited the morpheein concept—the ability of a homooligomeric protein to exist as an ensemble of physiologically significant and functionally different alternate quaternary assemblies—coming apart and changing shape so as to convert between forms [66]. Hysteresis, hydrophilic interactions, and a scaffold or chaperoning action (such as the chaperoning effect of galactose in the presence of the denaturants) are all characteristics of morpheein proteins [66]. However, as suggested in our earlier work [23], *β*-galactosidase exhibited moonlighting protein properties, peculiar to intrinsically unstructured proteins (IUPs) (but certainly not restricted to IUPs) where such proteins are able to fulfill more than one, apparently unrelated function using different sites [67]. This raises a question: is it possible for *β*-galactosidase to moonlight and at the same time morph by breaking down into varying secondary structures, at different times, so as to convert into entirely new forms with different functionalities? Could it also be possible that *β*-galactosidase performs its metabolic regulatory activities [62] via the morpheein pathway? These are questions we do not have immediate answers to. However, the current concepts of moonlighting and morpheeins call for review of the *C*-value paradox [68] to account for the wide array of protein functions running under a limited number of genes.
- Small molecules as effectors (e.g., as activators or inhibitors, see [69]) due to a greater accessibility of sites can effectively act as interference molecules to modulate the preference of one pathway over the other. This is seen from the pNPG and galactose actions. Our results suggest that pNPG bound to the denaturant-perturbed enzyme to effect catalysis while galactose bound to fold and subsequently effect catalysis. This is apparent in the different conformations induced at different [galactose] (this did not occur for different [pNPG]).

## 5. Conclusion

In conclusion, the possible implications of our findings in vivo in biological systems might include (1) that enzyme catalytic sites (as the binding funnel) and hydrophobic cores (as the folding funnel) could be modulated by the ligand to give rise to different stable conformations of the enzyme. (2) The binding mode, which drives folding, is determined by different forces arising from interaction sites. (3) Biological processes are carried out through binding, which respond to changes in the solvent environment. In different enzymes, the binding behaviour varies as a result of differences in structure/function and the environment. 

## Figures and Tables

**Table 1:** The values of *k*_+0_ and *k*_+0_′ for the corresponding concentrations of galactose.

	*k*_+0_	*k*_+0_′
3 M GdnHCl 0 mM Gal	0.0239	0.0111
5 mM	0.0305	0.0143
10 mM	0.0255	0.0292
20 mM	0.0192	0.0200
